# Efficacy of *Petasites hybridus* in migraine prophylaxis: the first real-world study

**DOI:** 10.3389/fneur.2026.1784624

**Published:** 2026-03-12

**Authors:** Raimundo Pereira Silva-Néto

**Affiliations:** Department of Neurology, Federal University of the Parnaíba Delta, Parnaíba, Brazil

**Keywords:** disability, efficacy, migraine, *Petasites hybridus*, prophylactic treatment

## Abstract

**Background:**

*Petasites hybridus* is a plant from the *Asteraceae* family used in migraine prophylaxis. Petasins and isopetasins, one of its constituents, act through antinociceptive, anti-CGRP, anti-inflammatory mechanisms and on calcium channels.

**Objective:**

This study aimed to evaluate the therapeutic efficacy of *Petasites hybridus* in the prophylaxis of episodic migraine (EM) and chronic migraine (CM).

**Methods:**

This was a single-center, retrospective, observational, uncontrolled, descriptive, and real-world study with 120 consecutive patients with EM or CM treated with *Petasites hybridus*.

**Results:**

One hundred and twenty patients (72 with EM and 48 with CM) were treated with *Petasites hybridus*, whose mean age was 35.4 ± 12.4 years, ranging from 18 to 60 years. Before treatment, the average frequency of headache for EM and CM was 6.0 ± 2.7 and 26.3 ± 5.1 days per month, respectively. After 12 weeks, there was a reduction in the number of days with headache, both in the EM and CM, respectively, to 2.6 ± 2.9 and 12.7 ± 8.6 (*p* < 0.0001). The reduction in headache attacks was greater than 50% in 59.2% of patients. There was a reduction in disability in both groups. Adverse events occurred in 28.3% of patients, including a bitter sensation in the mouth and/or eructation, lasting 6.4 ± 2.7 days and ranging from 2 to 14 days.

**Conclusion:**

*Petasites hybridus* was well tolerated and reduced the number of headache days per month by ≥50% in 60% of migraine patients within the first 12 weeks, providing a reduction in the degree of disability. Furthermore, *Petasites hybridus* (Petamig^®^) is free of pyrrolizidine alkaloids, making it appropriate and safe for prescription to patients.

## Introduction

Migraine treatment is based on patient education and abortive and prophylactic treatment of headache attacks. In Brazil, prophylaxis is indicated for patients who experience ≥3 headache attacks per month that interfere with their quality of life or who require symptomatic medication (1). However, in Europe and the USA, there are other recommendations based on different frequencies of monthly headache attacks (2, 3). Patients who experience less than one headache per month, but whose headache interferes with their routine activities, should also begin prophylactic treatment (4, 5). There are several categories of medications used in migraine prophylaxis, including beta-blockers, tricyclic antidepressants, calcium channel blockers, anticonvulsant neuromodulators, onabotulinumtoxinA, monoclonal antibodies, and herbal remedies such as *Petasites hybridus* (1).

*Petasites hybridus* is a plant from the *Asteraceae* family and has been used in traditional medicine for many centuries. However, in the last 25 years, it has been more commonly used in the prophylaxis of migraine, due to the action of petasins and isopetasins, one of its constituents (6, 7). It became a migraine prophylactic after several clinical studies proved its therapeutic efficacy (8–12).

Petasins and isopetasins act in migraine prophylaxis through four mechanisms of action. First, they have an antinociceptive effect by modulating the transient receptor potential ankyrin 1 (TRPA1) and the transient receptor potential vanilloid 1 (TRPV1), desensitizing TRP calcium channels (13, 14). Secondly, they exhibit an anti-CGRP effect, because by inhibiting the TRPA1 and TRPV1 ion channels, there is a reduction in the release of calcitonin gene-related peptide (CGRP) in meningeal afferents during headache attacks (15). Third, there is an anti-inflammatory effect, through the inhibition of phospholipase A2, lipoxygenase, and cyclooxygenase-2 activity, decreasing the production of inflammatory mediators involved in migraine attacks (6). Fourth, there is an effect on calcium channels, through direct interaction with high-voltage L-type calcium channels, which are crucial for antinociception (16).

Although several clinical studies have already demonstrated that *Petasites hybridus* is effective in migraine prophylaxis (8–12), further clinical studies are needed. Therefore, this first real-world study aims to evaluate the therapeutic effectiveness of *Petasites hybridus* in the prophylaxis of both episodic migraine (EM) and chronic migraine (CM).

## Methods

### Study design and patients

This was a single-center, retrospective, observational, uncontrolled, descriptive, and real-world study. The study population consisted of a non-random, convenience sample of consecutive patients diagnosed with EM or CM, treated at a headache clinic in Brazil for 2 years, who had been prescribed *Petasites hybridus* (Petamig^®^) in 50 mg capsules. There was no sample calculation. All patients were eligible according to the inclusion/exclusion criteria. Data for this study were collected from October 2023 to September 2025.

### Inclusion and exclusion criteria

Patients over 18 years of age, regardless of gender, diagnosed with migraine with a frequency greater than four headache days per month, by a headache expert, according to the ICHD-3 criteria (17) and being in continuous use of *Petasites hybridus*, as monotherapy, for a period of ≥3 months were included. The study excluded patients diagnosed with migraine concomitantly with another primary headache; with any secondary headache, who underwent treatment with botulinum toxin or monoclonal antibodies in the last 6 months, who started another prophylaxis 4 weeks prior to the onset of *Petasites hybridus*, pregnant women; and patients with liver and kidney diseases.

### Data collection

During the initial consultation, patients underwent a detailed medical history regarding the clinical characteristics of their headache, a neurological examination, determination of anthropometric indices (weight, height, and body mass index—BMI) using a standardized protocol, and hematological (blood count) and biochemical (blood glucose, lipid profile, renal and hepatic function) tests. Next, they answered the Migraine Disability Assessment Score (MIDAS) and Headache Impact Test (HIT-6) questionnaires to measure the degree of disability caused by migraine.

During the initial consultation, preventive treatment with *Petasites hybridus* was prescribed at a dose of 50 mg twice daily. Everyone received a headache diary to fill out, which included headache characteristics and detailed information about the use of symptomatic medications.

During headache attacks, patients were instructed to use a triptan in combination with a nonsteroidal anti-inflammatory drug, with a maximum frequency of 2 days per week. Those who reported excessive use of symptomatic medications in the past 3 months, both chronic migraine and episodic migraine with frequent headache attacks, characterized by taking two or more tablets per week, were instructed to abruptly discontinue the symptomatic medications they were using. They underwent a 5-day bridging course of prednisone at a dose of 40 mg/day.

### Evaluation of the use of *Petasites hybridus*

After 12 weeks of treatment with *Petasites hybridus*, patients were reassessed through a new medical history and headache diary to determine therapeutic effectiveness, a new response to the MIDAS and HIT-6 questionnaires to ascertain the improvement in the degree of disability and quality of life, reports of the presence or absence of adverse effects to determine the good tolerability of the medication, and repetition of hematological (blood count) and biochemical (blood glucose, lipid profile, renal and hepatic function) tests to assess the safety of the medication.

Treatment adherence was confirmed by telephone contact with patients 4 and 8 weeks after the prescription of *Petasites hybridus*. After 12 weeks, they returned for reassessment. Most improved with the 50 mg dose twice daily, but 15% (19/125) of patients showed no improvement after 8 weeks and were advised to increase the dose of *Petasites hybridus* to 50 mg three times daily.

The primary outcome of this study was the change from baseline over 12 weeks in the average number of headache days per month. Secondary outcomes included a 50% or greater reduction in the average number of headache days per month and a reduction in disability caused by migraine.

### Statistical analysis

The Statistical Package for the Social Sciences (SPSS^®^) version 18.2.2 was used for statistical analysis. Quantitative variables were expressed as mean, standard deviation, and minimum and maximum values, while qualitative variables were expressed as absolute and relative frequencies with 95% confidence intervals. The chi-square test with Yates correction, the Mann–Whitney test, and the Student’s *t*-test were used to compare the difference in means of paired samples. All tests were performed at a significance level of 0.05 for rejection of the null hypothesis.

## Results

One hundred and twenty-five patients (102 women and 23 men) were included in the study. After receiving a prescription for *Petasites hybridus*, five patients (four women and one man) did not return for their second medical appointment. They were excluded from the study. The sample studied consisted of 120 patients, with 60% (72/120) having EM and 40% (48/120) having CM. The mean age of the patients was 35.4 ± 12.4 years (95% CI 33.2–37.6), ranging from 18 to 60 years, of which 98 (81.7%) were women and 22 (18.3%) were men, which corresponded to a sex ratio of 4.5:1 female/male. Only four patients used a 5-day course of prednisone, but they were not excluded from the study because, although it could be a confounding factor, their exclusion did not change the significance of the study. BMI in patients with EM and CM was 23.4 ± 2.9 kg/m^2^ (95% CI 22.7–24.1) and 25.5 ± 3.8 kg/m^2^ (95% CI 24.4–26.6), respectively (*p* = 0.002) (Table 1).

**Table 1:** Distribution of sex and age according to diagnosis of 72 patients with episodic migraine and 48 with chronic migraine.

Variables	Comparison groups		*p*-value
	Episodic migraine	Chronic migraine	
Sex			0.885^a^
Female (*n*; %)	58 (80.6)	40 (83.3)	
Male (*n*; %)	14 (19.4)	8 (16.7)	
Age (years)			0.309^b^
Average	34.4 (12.9)	36.9 (11.4)	
95% CI	31.4–37.4	33.4–40.1	
Variation	18–57	18–60	
BMI (kg/m^2^)			0.002^b^
Average	23.4 (2.9)	25.5 (3.8)	
95% CI	22.7–24.1	24.4–26.6	
Variation	18.1–30.4	19.8–39.3	

CI, confidence interval; BMI, body mass index.

a *p*-value based on the chi-square test.

b *p*-value obtained using the Mann–Whitney test for the difference of means from unpaired samples.

Clinical and epidemiological characteristics of the 120 patients with migraine treated with *Petasites hybridus* are summarized in Table 2.

**Table 2:** Clinical and epidemiological characteristics of 120 patients with migraine treated with *Petasites hybridus*.

Pain characteristics	
Age at diagnosis (years; SD; variation)	35.4 ± 12.4 (18–60)
Latency to diagnosis (years; SD; variation)	15.8 ± 8.6 (2–25)
Time with headache >15 days/month (years; SD; variation)	6.1 ± 2.9 (1–8)
Body mass index (kg/m^2^; SD; variation)	24.2 ± 3.4 (18.1–39.3)
Headache days per month in the last 3 months (days/month; SD; variation)	13.9 ± 10.7 (4–30)
Episodic migraine	60.0
Chronic migraine	40.0
Quality of pain (%)	
Pulsatile	88.4
Dull/pressure	11.6
Pain intensity (%)	
Mild (VAS 1–4)	7.5
Moderate (VAS 5–7)	52.5
Severe (VAS 8–9)	22.5
Very severe (VAS 10)	17.5
Location of pain (%)	
Unilateral (65.7% on the left; and 34.3% on the right)	58.3
Bilateral	29.2
Holocranial/diffuse	12.5
Concomitant symptoms (%)	
Nausea and/or vomiting	65.0
Nausea and photophobia	19.2
Photophobia and phonophobia	10.0
Nausea and/or vomiting, photophobia and phonophobia	5.8
Aura symptoms (%)	
Presence	0.0
Absence	100.0
Trigeminal autonomic characteristics (%)	
Absent	88.3
Present (57.1% lacrimation; and 21.4% conjunctival hyperemia)	11.7
Medication overuse (%)	
Yes	23.3
No	76.7
Number of previous preventive treatments (%)	
None	32.5
One	12.5
Two	11.7
Three	22.5
>Four	20.8

SD, standard deviation; VAS, Visual Analogue Scale. Data are presented as percentages (%) and/or arithmetic mean ± standard deviation (range in parentheses).

The average frequency of headaches in the 3 months prior to treatment for EM and CM was 6.0 ± 2.7 (95% CI 5.38–6.62) and 26.3 ± 5.1 (95% CI 24.86–27.74) days per month, respectively. After 12 weeks of treatment, there was a reduction in the number of headache days per month, for both EM and CM, to 2.6 ± 2.9 (95% CI 1.93–3.27) and 12.7 ± 8.6 (95% CI 10.27–15.13), respectively (*p* < 0.0001). There was no change in BMI. The disability assessment instruments (MIDAS and HIT-6) demonstrated a reduction in all scores when compared to baseline, for both EM and CM, as shown in Table 3.

**Table 3:** Comparison between baseline and after 12 weeks of treatment with *Petasites hybridus* in 120 patients with migraine.

Variables	Comparison groups		*p*-value*
	At baseline	After 12 weeks	
Episodic migraine (mean; SD; CI)			
Frequency of headache attacks (days per month)	6.0 ± 2.7 (5.38–6.62)	2.6 ± 2.9 (1.93–3.27)	<0.0001
Body mass index	23.4 ± 2.9 (22.73–24.07)	23.3 ± 2.8 (22.65–23.95)	0.530
MIDAS	14.4 ± 6.5 (12.90–15.90)	3.5 ± 2.4 (2.95–4.05)	<0.0001
HIT-6	57.1 ± 8.6 (55.11–59.09)	38.2 ± 6.6 (36.55–39.85)	<0.0001
Chronic migraine (mean; SD; CI)			
Frequency of headache attacks (days per month)	26.3 ± 5.1 (24.86–27.74)	12.7 ± 8.6 (10.27–15.13)	<0.0001
Body mass index	25.5 ± 3.8 (22.42–24.58)	25.5 ± 3.8 (22.42–24.58)	0.183
MIDAS	21.8 ± 4.3 (20.58–23.02)	8.0 ± 2.5 (7.24–8.76)	<0.0001
HIT-6	57.5 ± 8.4 (55.12–59.88)	39.8 ± 8.4 (37.26–42.34)	<0.0001

SD, standard deviation; CI, confidence interval; MIDAS, Migraine Disability Assessment Score; HIT-6, Headache Impact Test. ^*^p-value calculated using the Student’s *t*-test.

After 12 weeks of treatment with *Petasites hybridus*, there was a reduction in headache attacks of ≥50% in 59.2% (71/120) of patients. In patients with EM and CM, this reduction was 63.9 and 52.1%, respectively, with no statistical difference (Table 4).

**Table 4:** Reduction ≥50% in headache attacks in migraine patients after 3 months of treatment with *Petasites hybridus*.

Variables	Patients with migraine (*n*; %)	Comparison groups		*p*-value
		Episodic migraine (*n*; %)	Chronic migraine (*n*; %)	
Reduction in headache attacks ≥50% after 3 months				0.272
Yes	71 (59.2)	46 (63.9)	25 (52.1)	
No	49 (40.8)	26 (36.1)	23 (47.9)	

Adverse effects possibly related to the use of *Petasites hybridus* occurred in 28.3% of patients and were the following gastrointestinal disorders: bitter taste in the mouth and belching (15%), bitter taste in the mouth or belching (10%), nausea (1.7%), abdominal pain (0.8%) and diarrhea (0.8%), with a mean duration of 6.4 ± 2.7 days and ranging from 2 to 14 days (Table 5).

**Table 5:** Adverse effects considered possibly related to the use of *Petasites hybridus* and their duration in 120 patients with migraine.

Adverse effects	Frequency (*n*; %)	Duration (days; SD; variation)
Absence	86 (71.7)	—
Presence	34 (28.3)	6.4 ± 2.7 (2–14)
A bitter sensation in the mouth and eructation	18 (15.0)	7.1 ± 2.5
A bitter sensation in the mouth	8 (6.7)	6.6 ± 2.5
Eructation	4 (3.3)	6.3 ± 2.5
Nausea	2 (1.7)	4.0 ± 1.0
Diarrhea	1 (0.8)	3.0 ± 0.0
Abdominal pain	1 (0.8)	2.0 ± 0.0

SD, standard deviation.

The hematological (blood count) and biochemical (blood glucose, lipid profile, renal and hepatic function) tests performed during the initial consultation and after 12 weeks were all normal.

## Discussion

Migraine is a chronic neurological disease that affects millions of people worldwide, both during the ictal and interictal phases. It is one of the main causes of disability and impacts quality of life (18, 19). For this reason, prophylactic treatment is necessary to reduce headache attacks and associated symptoms, improve quality of life, and prevent progression to CM (20). There are several treatments available for migraine prophylaxis that, despite proven efficacy, are not always well tolerated or have a high cost, restricting access to their use (21). Many studies have already shown the efficacy and safety profile of various migraine prophylaxis agents (22), including *Petasites hybridus* (8–12).

In this first real-world study, patients treated with *Petasites hybridus* experienced a reduction in the number of headache days per month, for both EM and CM. There is no other real-world study to compare with our study. There are only double blind, randomized, placebo-controlled studies that have demonstrated the efficacy of *Petasites hybridus* in migraine prophylaxis (8–10), as summarized in Table 6.

**Table 6:** Analysis of three clinical studies in adult patients with migraine who were treated with *Petasites hybridus*.

Variables	Published studies		
	Grossman and Schmidramsl (8)	Diener et al. (9)	Lipton et al. (10)
Type of study	Double-blind, randomized, and placebo-controlled	Double-blind, randomized, and placebo-controlled	Randomized, parallel groups and placebo controlled
Number of patients	60	60	202
Intervention	50 mg twice a day vs. placebo for 12 weeks	50 mg twice a day vs. placebo for 12 weeks	75 mg twice a day vs. 50 mg twice a day vs. placebo twice a day for 16 weeks
Results	The migraine group reduced the number of headache days from 3.3 to 1.7 (*p* < 0.05), an improvement of 60%	The migraine group reduced the number of headache days from 3.4 to 1.8 (*p* = 0.002); and in placebo, from 2.9 to 2.6 (ns)	Reduction in the number of headache days by 48% with the 75 mg twice-daily dose (*p* = 0.001); by 36% with the 50 mg twice-daily dose (*p* = 0.127); and by 26% with placebo
Conclusions	Patients with migraine may benefit from prophylaxis with *Petasites hybridus*	*Petasites hybridus* was well tolerated and proved to be effective in migraine prophylaxis	*Petasites hybridus* 75 mg twice a day is more effective than placebo and is well tolerated in migraine prophylaxis

As in previous studies, in our study there was also a reduction in headache attacks >50% in 60% of patients after 12 weeks of treatment with *Petasites hybridus* as monotherapy, especially in patients with EM (10). This reduction in headache attacks was similar to clinical studies conducted in patients who used monoclonal antibodies (23). Furthermore, a randomized, double-blind, controlled study showed that *Petasites hybridus* had comparable efficacy to propranolol and topiramate (24).

At baseline in our study, migraine patients had a moderate to severe degree of disability. These data are consistent with previously published data that showed migraine to be the second most disabling condition in the world, especially chronic migraine (CM), which is associated with greater disability (19). After 12 weeks of treatment with *Petasites hybridus*, in addition to a reduction in headache days per month, there was also a reduction in the degree of disability, characterized by a reduction in MIDAS and HIT-6 scores, for both CM and EM (*p* < 0.0001). Possibly, the therapeutic efficacy of *Petasites hybridus* in migraine prophylaxis demonstrated in this study may contribute to preventing the progression from EM to CM, which occurs in approximately 2.5% of people with migraine (19, 20).

Based on findings from previously conducted randomized clinical trials, the guidelines from the American Academy of Neurology and the American Headache Society considered *Petasites hybridus* effective in preventing migraine, with a level A evidence for the prophylactic treatment of EM. Surprisingly, among the drugs recommended for migraine prevention, *Petasites hybridus* was the only herbal medicine with “level A” evidence of efficacy and safety (25, 26).

The choice of migraine prophylaxis should be individualized, taking into account the potential for adverse effects, drug interactions, and comorbidities of each patient (27). Oral medications available for the prophylactic treatment of migraine have robust clinical efficacy, but numerous adverse reactions have been reported (28, 29), which represent one of the main reasons for patients to discontinue preventive treatment (30, 31).

In this study, all patients required prophylactic treatment. The decision-making process regarding medication choice was shared with the patient, who was able to express their preferences about the treatment process. The various categories of migraine prophylaxis, especially oral medications, were presented to the patients, highlighting their effectiveness, cost, and adverse effects. Most patients preferred a herbal remedy, due to the belief that this treatment is non-pharmacological. Similarly, recent studies have highlighted the preference of migraine patients for treatments formulated in tablet or non-pharmacological form (32, 33).

In previous studies, *Petasites hybridus* was well tolerated, and adverse events were of mild to moderate intensity without any clinical relevance, with gastrointestinal disturbances predominating, especially eructation. They occurred at comparable frequencies in all groups (8–10). In this study, the adverse effects related to the use of *Petasites hybridus* were only gastrointestinal, of mild intensity, transient, and did not cause any patient to discontinue their treatment. *Petasites hybridus* did not interfere with appetite, as the mean BMI at baseline and after 12 weeks did not change (Table 5).

Plants in the *Asteraceae* family produce pyrrolizidine alkaloids that have hepatotoxic, mutagenic, and carcinogenic effects (6, 34). Therefore, it is recommended that patients use *Petasites hybridus* that are certified and labeled as free of these alkaloids. In our study, the treatment was carried out with *Petasites hybridus* (Petamig^®^), whose standardized extract used in its production is obtained by supercritical CO_2_ extraction (35). Due to this extraction method, Petamig^®^ is free of pyrrolizidine alkaloids, with less than 0.08 ppm of the alkaloid in the nutraceutical (36) and is appropriate and safe to prescribe to patients (36–38).

In this study, liver and kidney function were monitored, as recommended by some authors (39). All patients presented normal renal and hepatic function before and after treatment with *Petasites hybridus*. Similarly, a narrative review of randomized, double-blind, placebo-controlled studies investigated the safety of *Petasites hybridus* in migraine prevention and found no relevant alteration in liver function. Liver function tests were performed on primary human hepatocyte cultures and did not show hepatotoxicity with therapeutic doses of *Petasites hybridus* and with concomitant migraine medications (14).

### Study limitations

This study had some limitations, such as a retrospective design; not being double-blind, randomized, and placebo-controlled; potential selection bias due to the sample being a convenience sample from a single center; relatively small sample size; lack of control for the potential confounding effect of prednisolone use; a follow-up period of only 12 weeks; and a scarcity of previous clinical studies or systematic reviews. However, we believe that the aforementioned limitations did not interfere with the generalizability of the results. On the other hand, the same neurologist was responsible for the initial assessment and the reassessment after 12 weeks, which is a strong point of the study.

## Conclusion

*Petasites hybridus* was well tolerated and reduced the number of headache days per month by ≥50% in 60% of migraine patients within the first 12 weeks, providing a reduction in the degree of disability. Furthermore, *Petasites hybridus* (Petamig^®^) is free of pyrrolizidine alkaloids, making it appropriate and safe for prescription to patients.

## Data Availability

The original contributions presented in the study are included in the article/supplementary material, further inquiries can be directed to the corresponding author.
